# New Biocompatible Nanohydrogels of Predefined Sizes for Complexing Nucleic Acids

**DOI:** 10.3390/pharmaceutics15020332

**Published:** 2023-01-19

**Authors:** Lakshmanan Eswaran, Gila Kazimirsky, Gerardo Byk

**Affiliations:** Laboratory of Nanobiotechnology, Department of Chemistry, Bar-Ilan University, Ramat Gan 52900, Israel

**Keywords:** self-assembly, polymerization, cationic nanohydrogels, non-viral gene delivery

## Abstract

The advent of protein expression using m-RNA applied lately for treating the COVID pandemic, and gene editing using CRISPR/Cas9 technology for introducing DNA sequences at a specific site in the genome, are milestones for the urgent need of developing new nucleic acid delivery systems with improved delivery properties especially for in vivo applications. We have designed, synthesized, and characterized novel cross-linked monodispersed nanohydrogels (NHG’s) with well-defined sizes ranging between 50–400 nm. The synthesis exploits the formation of self-assemblies generated upon heating a thermo-responsive mixture of monomers. Self-assemblies are formed and polymerized at high temperatures resulting in NHGs with sizes that are predetermined by the sizes of the intermediate self-assemblies. The obtained NHGs were chemically reduced to lead particles with highly positive zeta potential and low cell toxicity. The NHGs form complexes with DNA, and at optimal charge ratio the size of the complexes is concomitant with the size of the NHG’s. Thus, the DNA is fully embedded inside the NHGs. The new NHGs and their DNA complexes are devoid of cell toxicity which together with their tunned sizes, make them potential tools for gene delivery and foreign protein expression.

## 1. Introduction

The synthesis of monodispersed nanoparticles with well tunned sizes is a complex process of great interest since size and dispersity determine the feasibility of different applications. Their generation has been tackled mostly by synthesizing nanoparticles made of inorganic materials such as silica, titanium oxide, iron, gold, and silver or by more complex mixtures of metals such as quantum dots (for example CdSe/ZnS) whose precursors can drive a controlled formation of monodispersed particles. These materials are known as having significant cell toxicity and the tendency to leak out from the nanoparticles [1]. Therefore, most of the available approaches adapt these materials to different biological applications by developing further coating methods that render them more biocompatible. Coatings are composed of organic materials, mainly polymers that are adsorbed, anchored, or polymerized on the surface of the inorganic nanoparticles resulting in composite particles with lower toxicity and better bioavailability [2]. In summary, the syntheses of these materials are tedious and time-consuming.

During the last few years, we have considered the possibility of generating new types of cross-linked nanoparticles composed of polymeric hydrogels. Nanohydrogels (NHGs) have gained significant attention in recent years for drug delivery and tissue engineering, owing to their peculiar properties that combine the characteristics of hydrogel systems (e.g., high water content) with a very small size (nanometric dimension). Their properties enable reaching the smallest capillary vessels, not accessible to macroscale hydrogels, and penetrate tissues either through the paracellular or transcellular pathways. The size and surface properties of NHGs can be manipulated to avoid rapid clearance by phagocytic cells, and so extending their circulation times, allowing both passive and active drug targeting. Hydrogel-based delivery devices can be used for oral, rectal, ocular, epidermal, and subcutaneous applications [3]. Hydrogel NPs are used as scaffolds for drug delivery since they start decomposing after administration with a concomitant release of their drug cargo. Paradoxically, good biodegradability brings about low toxicity, and increased bio-stability of particles likely infers toxicity [4].

The size and composition of hydrogel NPs have been tuned using block copolymers composed of hydrophilic and hydrophobic segments that can form self-assemblies under specific conditions and eventually be polymerized for their stabilization. For example, tri-block copolymers of type A-B-A composed of PEO-PPO-PEO (Pluronic surfactant) or the reverse form PPO-PEO-PPO have been used for tailoring self-assemblies for controlled drug delivery [5]. On the same basis, the inclusion of cross-linkable functions in the block-copolymers allows not only the formation of NP self-assemblies, but also their cross-linking through these functions. One of the early cases of this kind of approach was the synthesis of polymersomes which are liposome-like polymeric vesicles composed of block copolymers [6]. Star-like particles can also be obtained with a block-copolymer of type A-B, [7] onion–like micelles by mixing tri-block A-B-A type and di-block A-B type copolymers, [8] and, in two very recent applications, flower-like NPs were obtained using tri-block-copolymers of A-B-A type [9] and a combination of star and flower shaped micelles were obtained by self-assembling tri-block A-B-A type and di-block A-B type copolymers with an outer hydrophilic shell exposed to the aqueous phase and a hydrophobic inner core encapsulating a hydrophobic drug [10]. Finally, in a very peculiar study, monomers such as acrylonitrile (ACN) and N-isopropylacrylamide (NIPAAM) were self-assembled prior to polymerization using sodium dodecyl sulfate (SDS) as a micellar scaffold. According to the report, the more hydrophobic ACN penetrates the micelle and forms the core of the particle at room temperature, while the NIPAAM remains outside. According to these studies, raising the temperature has a particular effect on NIPAAM which becomes more hydrophobic as hydrogen bonding with water is exchanged by intermolecular hydrogen bonding between molecules of NIPAAM monomers through the amide bonds and hydrophobic interactions between i-propyl groups of different molecules. The increased hydrophobicity of monomeric NIPAAM enables its penetration into the micelle scaffold. The addition of initiator brings about the formation of a core-shell NP with most of the polyacrylonitrile in the core and most of the PNIPAAM in the shell [11]. Recently, we have developed a new concept for the generation of monodispersed NHGs that exploits the importance of this interesting approach for obtaining not only specific sizes of NHGs but a panel of sizes ranging from 20 to 500 nm using different ratios of NIPAAM in the starting mixture [12]. The most important physical parameter for designing chemo-stable biocompatible NPs is their size which avoids not only renal excretion but also recognition and uptake by the reticuloendothelial system (RES) which can shuttle the NPs out of the circulation to the liver, spleen, or bone marrow [13]. The optimal size depends on the type of tissue that must be reached. For NPs to be suitable for IV administration (circulation) they must be smaller than capillary vessels (5–10 µm). Moreover, the NPs must remain in the bloodstream long enough to reach and recognize their site of action. Finally, they must not aggregate since aggregation presents a risk of embolism due to capillary occlusion [14]. The opsonization or removal of nano-particulate drug carriers from the body by the mononuclear phagocytic system (MPS), also known as the reticulo-endothelial system (RES), is a major obstacle to in vivo compatibility of NPs. The macrophages of the MPS can remove uncoated NPs from the bloodstream within seconds of intravenous administration, rendering them ineffective as site-specific drug delivery devices [15]. These macrophages cannot directly identify the NPs themselves, but rather recognize specific opsonin proteins (blood serum components) which adsorb to the surface of the particles [16]. In numerous works, it has been shown that one well-known compound that can reduce MPS recognition of NPs is polyethylene glycol (PEG), also known as polyethylene oxide (PEO). In both drug delivery and imaging applications, the addition of PEG to NPs either by grafting methodology or copolymerization of PEG with other monomers reduces RES uptake and increases circulation time, compared to uncoated counterparts [17,18]. Thus, PEG-coated NPs have a “stealth” behavior [19,20,21]. Another material, poly-N-isopropyl acrylamide (PNIPAAM), when combined with PEO-based polymers, is especially interesting for biological and medicinal applications [22,23]. PEG-based polymers have also been used in the field of peptide/organic solid-supported synthesis with significant success (PEGA [24] and Tentagel [25] solid supports). PEGA microspheres of about 500 µm were applied to both synthesis and biological assays based on seeding cells on these large microspheres [25]. Since cross-linked PEG microspheres are hydrogels permeable to physiological solutions, they can be used as a scaffold for synthetic tissue engineering or binding assays. However, NPs containing PEG used for drug delivery, include in their backbone biodegradable functions such as esters that make them sensitive to chemical and enzymatic degradation necessary for releasing a loaded drug [26,27]. In addition to biocompatibility, if NPs are suitable for organic synthesis on their surface their applicability as biosensors will raise significantly. Therefore, cross-linking within the material of the NPs and exclusion of labile organic functions are mandatory for obtaining bio-chemically stable NPs. In the few cases where PEG-based particles are cross-linked, they are obtained as poly-dispersed mixtures ranging from 300 to 700 µm, which is very far from the size needed for live cells or in vivo applications [28].

Our recently presented methodology for obtaining cross-linked and size-tuned polyethylene glycol NPs ranging between 20 to 500 nm with the qualities of being mono-dispersed, not ready degradable, and non-toxic remains unique in the field. In the current work, we further developed a new generation of NHGs by the introduction of new monomers and methodologies that generate NHGs suitable for complex nucleic acids as potential tools for gene delivery and foreign protein expression.

## 2. Materials and Methods

### 2.1. Materials

Polymerizations were performed in a DAIHAN scientific shaking water bath. Jeffamine^®^ ED-2003 (Mn = 1900), acryloyl chloride, acrylonitrile, and polyvinylpyrrolidone (PVP_360,000_) were purchased from Sigma-Aldrich (St. Louis, MI, USA), N-isopropylacrylamide (NIPAM) (99.0%) was purchased from Acros organics Co., Ltd. (St. Louis, MI, USA), and N, N’-methylenebisacrylamide (BIS) (97.0%) was purchased from Alfa Aesar Co., Ltd., Haverhill, UK. Cellulose Ester Spectra/Pore 1000 kDa cut-off molecular weight dialysis membrane was purchased from spectrum laboratories, Inc., Rancho Dominguez, CA, USA. Hydrodynamic sizes, size distributions, and zeta potential measurements were determined using a Zetasizer 3000 HSA (Malvern Instruments Ltd., Malvern, UK) operating with a 4 mW HeNe laser (632.8 nm), a detector positioned at a scattering angle of and a temperature-controlled jacket for the cuvette. The FTIR spectra were recorded on Thermo Nicolet iS10 with a smart omni transmission spectrometer. TGA was carried out using a Perkin Elmer Clarus 680/Clarus SQ 8C thermogravimetric analyzer. AFM was carried out using Bruker, AXS. XTT Assay Kit was purchased from Biological Industries, Beit Haemeq, Israel. Humidified incubator (Water-Jacketed, US Autoflow Automatic CO_2_ Incubator manufactured by NuAire, Inc., Plymouth, MA, USA) was used for cell culture.

### 2.2. Synthesis of NHG’s Containing Highly Reducible Groups

Briefly, 5 mg of BIS (0.03 mmol), 5 mg of PVP_360,000_, 170 mg of NIPAAM (1.66 mmol), 60 mg acrylonitrile (11 mmol) and an appropriate amount of (Acr)1.1Jeffamine1900 for obtaining the desired NHG’s size (200 mg for 400 nm, 400 mg for 200 nm and 500 mg for 50 nm), were introduced into a 20 mL scintillation vial and fully dissolved in 8 mL DDW. The vial content was flushed with nitrogen and shaken at 73 °C for 1 h in a shaking water bath. Then, a solution of KPS (5 mg, 0.018 mmol) in 2 mL DDW was added to the reaction mixture, and the polymerization process was allowed to continue at 73 °C for 23 h. The resulting NHG’s dispersion was allowed to cool to room temperature and dialyzed against 25 L DDW for 1 week. The water was changed twice per day. Aliquots of purified NHG’s dispersion were lyophilized to determine polymerization yield.

### 2.3. Reduction of NHG’s (Method A)

The centrifuged polymeric NHG’s (160 mg) and 319 mg boric acid (5.16 mmol) was poured into a glass pressure tube. A total of 607 µL of trimethyl borate (5.16 mmol) was added followed by the addition of 17 mL 1 M borane-THF complex (17 mmol). After the cessation of hydrogen evolution, the tubes were sealed and kept in an oil bath at 65 °C for 72 h. The NHG’s were then centrifuged and washed with MeOH (10 mL × 2). The obtained precipitate was mixed with methanol (8 mL) and piperidine (2 mL), transferred to a reactor, and heated to 65°C for 20 h under pressure to destroy the borane complexes. Following the decantation of the piperidine-borane solution, water was added, and the suspension was centrifuged and washed with distilled water (10 mL × 3). The reduced NHGs were dialyzed against 25 L DDW for 2 days. The water was changed twice per day.

### 2.4. Reduction of NHG’s (Method B)

#### Preparation of the Magnetic Embedded NHG’s Matrix

A total of 25 mL of 50 nm NHG’s aqueous dispersion (about 200 mg NHG) were added to a 1 L three-necked bottom round flask and cooled in a water bath under nitrogen for 45 min under vigorous stirring. A total of 200 mg (0.8 mmol) FeCl_3_.6 H_2_O and 140 mg (0.6 mmol) FeCl_2_.4 H_2_O were dissolved in 3 mL DDW. Then the iron solution was added to the flask containing NHGs. A light-yellow color mixture was formed. The ice bath was removed, and the flask was continuously evacuated under a vacuum with the mixture kept stirring until no further foaming in the mixture was observed. The evacuation was stopped, and the flask was immersed in a pre-heated water bath at 85 °C. 5 mL of ammonium hydroxide (30%) was added. The reaction mixture turned gradually brown. The mixture was kept stirring at 85 °C for 1.5 h, then cooled to room temperature. The resulting magnetic matrix was washed extensively with water (20 mL × 5), DMF (20 mL × 5) methanol (20 mL × 5) and diethyl ether (20 mL × 5) using an external magnetic field for separation to obtain 350 mg of magnetic matrix embedded 50 nm NHG’s. The magnetic matrix embedded NHGs were totally retained after applying a magnetic field during few minutes (using an N50 magnet). The magnetic matrix-embedded NHG’s were analyzed by TGA.

### 2.5. Reduction of Magnetic Embedded NHG’s

A total of 40 mg of magnetic NHG’s (50 nm) matrix and 79 mg boric acid (1.27 mmol) were taken in a reactor. A total of 150 µL of trimethyl borate (1.27 mmol) was added followed by the addition of 4.25 mL 1 M borane-THF complex (4.25 mmol). After the cessation of hydrogen evolution, the reactor was capped tightly and kept in an oil bath at 65 °C for 96 h. The matrix was then centrifuged and washed with MeOH (20 mL × 5), DMF (20 mL × 5), and water (20 mL × 5) using magnetic susceptibility for separation. The resulting matrix was kept in 10 mL H_2_O.

### 2.6. Dissolving the Magnetic Matrix for Releasing the NHG’s

The matrix (40 mg) is separated from the water and 8 mL of HCl (5 M) was added and stirred for 6 h. The full dissolution of the matrix and regeneration of NHG’s were monitored by DLS. Then, an excess of EDTA tetrasodium salt (200 mg, 0.52 mmol) was added to the acidic dispersion of NHG’s containing iron salt ions. The resulting mixture was neutralized slowly by adding 30% NH_4_OH to pH = 6. Then, the mixture was sonicated for several minutes and dialyzed against 25 L water for 2 days by replacing the water twice a day to obtain reduced NHG’s.

### 2.7. DLS and Zeta Potential

Hydrodynamic sizes, and size distributions of NHG’s were measured using diluted NHGs (50 µg/3 mL). For the zeta potential measurements, 2 mL of (1 mg/mL) NHG’s (pH = 7.4) dispersion was injected into the zetasizer 3000 HSA pump. Three measurements consisting of 10 sub runs were performed for each sample at 25 °C and 45 °C.

### 2.8. Plasmid Preparation

The plasmids used in this work were Monster Green^®^ Fluorescent Protein GFPphMGFP Vector. It contains the open reading frame for the Monster Green^®^ Fluorescent Protein cloned into a mammalian expression vector. The Monster Green^®^ Fluorescent Protein is encoded by an improved synthetic version of the green fluorescent protein gene originally cloned from Montastrea cavernosa (Great Star Coral) and the synthetic gene (hMGFP) expresses a 26 kDa protein (Source: www.promega.com/tbs and CatLog number: E6421). The plasmid DNA was amplified in E. coli and purified according to the supplier’s protocol (Promega, Madison, WI, USA). The quantity and quality of the purified plasmid DNA were assessed by optical density at 260 and 280 nm and by electrophoresis in 0.8% agarose gel.

### 2.9. Preparation of Polyplexes for Size and Zeta Potential Measurement

Polyplexes were prepared by mixing various weight ratios of reduced NHG’s and pDNA (40:1, 20:1, 10:1, 5:1, 1:1 and 1:10). A total of 500 µg/mL NHG’s solution was mixed with 2.5 mL DDW and then added into 20 µL of 12.5 µg pDNA solution to obtain a 40:1 ratio. In a similar way, 12.5 µg pDNA was used for all the other ratios with the appropriate amount of NHG’s. Then the samples were mixed by gently pipetting and incubated at room temperature for 20 min to ensure polyplexes formation.

For the zeta potential: 1 mg/mL NHG’s solution was mixed with 1 mL PBS (pH–7.4) and then added into 1 ml of diluted pDNA (25 µg) solution to obtain a 40:1 ratio. In the same way, 25 µg pDNA was used for all other ratios with appropriate amounts of NHG’s. The samples were mixed by gently pipetting and incubated at room temperature for 20 min. Then the polyplexes size and zeta potential were measured by DLS.

### 2.10. Fourier-Transform Infrared Spectroscopy (FTIR)

The spectra of reduced and non-reduced NHG’s (400, 200 and 50 nm) were acquired (32 scans per sample or background) in the range of 4000–500 cm^−1^ and were corrected using the background spectrum of air. The analyses were carried out at room temperature. For measurement, a lyophilized sample of NHG’s was dissolved with paraffine and placed onto the surface of the NaCl glass.

### 2.11. Thermogravimetric Analysis (TGA)

Samples of 50 nm reduced NHG’s (~1 mg) were heated from 30 to 850 °C at a heating rate of 20° min^−1^ in a nitrogen atmosphere. An empty magnetite matrix was used as control.

### 2.12. Atomic Force Microscopy (AFM)

#### 2.12.1. Method (A)

The AFM of the NHG’s sample was prepared by a simple spin coating method on a silicon wafer substrate using (1 mg/mL NHG’s, 50 µL) of NHG’s. The spin coating timer was adjusted for 30 s at 2000 RPM. After spin-coating samples were thoroughly dried out with nitrogen and analyzed.

#### 2.12.2. Method (B)

The AFM of the polyplex complexes was coated on a mica substrate as a drop-casting technique. Polyplex complexes made of 10 µg/40 µL NHG’s and 0.5 µg/40 µL pDNA were mixed and incubated for 20 min. The 80 µL of polyplex complexes were coated on the substrate and left there for 5 min. Then the polyplex-coated substrate was dried out with nitrogen and analyzed.

### 2.13. Gel Electrophoresis

To prepare complexes, 0.5 μg of pGFP (20 µL) was incubated for 20 min with varying amounts of NHG’s (20 µL). The complexes were mixed with 5 µL of loading dye and were loaded on a 0.8% agarose gel (400 mg) containing 5 µL Gel-Red. The electrophoresis study was performed at 100 V for 40 min in a TBE buffer medium and then the bands were monitored using a UV trans-illuminator.

### 2.14. Cell Culture

HEK-293T and HeLa cells were cultured in DMEM medium and supplemented with 10% fetal bovine serum (FBS, Sigma-Aldrich, New York, NY, USA), 100μg/mL streptomycin, and 100 U/mL penicillin at 37 °C in 5% CO_2_.

### 2.15. Cell Proliferation Assays

The cell proliferation of the cells was analyzed by XTT assay. The cells with their media were seeded in 96-well flat-bottom plates at a density of 8 × 10^3^ cells per well and cultured in a humidified incubator at 37 °C for 24 h. Then, the media was removed by aspiration and 90 μL of fresh media was added. The cells were treated with 20 µg of naked NHGs in 100 μL DMEM, polyplexes at ratio NHG/DNA 40:1 (20 μg NHG/0.5 μg DNA), 6 nmol lipoplexes in 100 μL DMEM, 0.5 µg DNA in 100 μL DMEM, and 2% triton in 100 μL DMEM. Briefly, a dose of polyplexes/lipoplexes in media was added to each well (in quadruplicate) and incubated for 48 h. One row was used as control cells without the addition of polyplexes/lipoplexes, and one row containing only the appropriate medium was used for blank absorbance readings. Subsequently, 50 μL of XTT reagent (with initiator) was added to the cells and incubated for 4 h. The cells’ absorbance was read at 570 nm in a TECAN microplate reader. Cell proliferation % = (Isample/Icontrol) × 100, where Isample is the absorbance of NHG’s treated wells and Icontrol is the absorbance of control wells without NHG’s treatment. These experiments were repeated twice.

## 3. Results and Discussion

### 3.1. Synthesis and Characterization of Nitrile Containing Nanohydrogels

**Figure 1:**
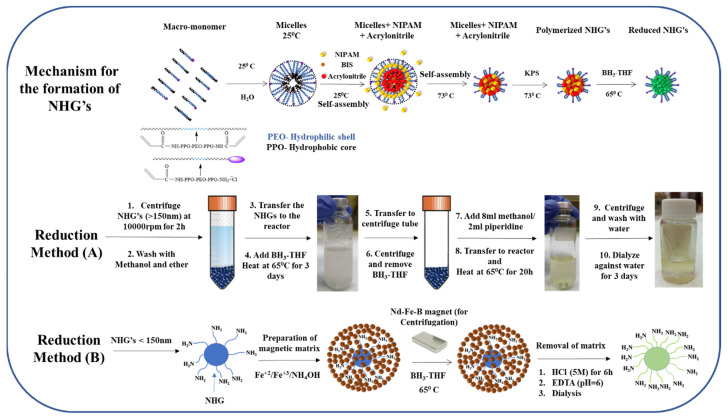
The mechanism for formation of NHG’s and reduction methods.

NIPAM, acrylonitrile (ACN), surfactant (PVP), cross-linker (BIS) and acrylated jeffamine macro-monomers were mixed at different ratios in the presence of initiator KPS (see Table 1). DLS analysis of the starting mixtures suggests that macro-monomers generate micelles at room temperature and the monomeric NIPAAM does not significantly change the size of these assemblies because it remains in solution. According to earlier studies, the NIPAAM becomes more hydrophobic when heated. The hydrophobic PPO segments of the block macromonomer collapse together with the relatively hydrophobic acrylonitrile (ACN)^11^ to produce a well-defined micelle with a hydrophobic core formed by ACN, PPO, and NIPAAM and a corona formed by PEO chains and free amino groups. Upon addition of initiator at 73 °C, polymerization starts, and stable cross-linked NHGs are generated with sizes corresponding to the intermediate micelles. Figure 1 (see upper panel) discloses the proposed mechanism of NHG’s formation. We observe that at constant amounts of NIPAM and ACN, varying the amount of macromonomer results in the generation of different NHG’s sizes. The lower the amount of macromonomer, the larger the NHG’s. Sizes are well monodispersed with a polydispersity index between 0.13 to 0.33 as measured by DLS (Table 1). The current NHG’s are new since they include in their preparation ACN which was absent in previous works. The presence of ACN groups in the NHG’s allows for obtaining NHG’s with a high number of free amino groups upon reduction. After the obtention of the nitrile containing NHG’s, they are submitted to chemical reduction. The reduction reactions are performed using Borane:THF. A difficulty for this reaction is the presence of water as solvent of the NHG which is incompatible with the use of Borane:THF. Two different procedures were established depending on the size and nature of the NHG’s (see middle and lower panels in Figure 1).

**Figure 2:**
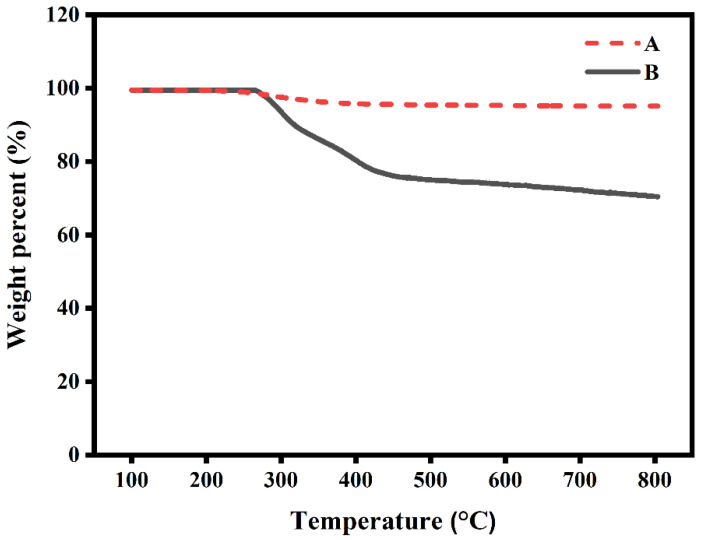
TGA curves of the pure magnetic matrix (A) and magnetic matrix embedded 50 nm NHG’s (B).

NHG’s of 400 and 200 nm could be precipitated easily using a centrifuge. Thus, for these cases, the solvent was easily exchanged from water to THF upon centrifugation and exhaustive washings to remove water (see method A in materials and methods). The smaller NHG’s of 50 nm presented a challenge since they do not precipitate upon centrifuge. To reduce these NHG’s we choose to use a method we have developed in our previous investigations for peptide synthesis on nanoparticles [29]. The method is based on the embedment of the NP’s into a magnetic matrix of magnetite. Briefly, magnetite is generated in the presence of the NHG’s that are embedded into the magnetite during the preparation of the magnetite. The obtained matrix-embedded NHG’s can be easily washed to exchange the solvent taking advantage of the magnetic susceptibility of the matrix. Thermo-gravimetric analysis (TGA) provided direct evidence for the presence of NHG’s in the magnetic matrix as previously demonstrated. A pure magnetic matrix has a very small weight loss change upon heating up to 800 °C [29]. At the same temperature range, the magnetic matrix embedded NHG’s showed a significant weight loss (Figure 2). The total weight loss for 50 nm embedded NHG’s was approximately 30%, which represents the relative quantity of NHG’s within the magnetic matrix. The reaction is then carried out using Borane:THF. Interestingly, even in the presence of magnetite that can be itself reduced; we observe an efficient reduction of the 50 nm NHG’s (see method B in materials and methods).

Results in Table 1 show NHG’s sizes as obtained by DLS before and after reduction. No significant size change was observed when comparing original NHG’s with their reduced counterparts. Size analysis at two different temperatures (25 °C and 45 °C) indicate similar thermo-responsivity before and after reduction. The sustained thermo-responsivity indicates that the PNIPAM component of the NHG’s is mostly unreduced due to steric hindrance at the proximity of the carbonyl groups induced by the i-Propyl nitrogen substitutions. This probably maintains the typical thermo-responsivity observed for PNIPAM. We note the clear reduction of the nitrile groups as seen by IR analysis (see details below).

### 3.2. Zeta Potentials

Zeta potential of the new NHG’s is crucial for the proposed applications since the starting NHG’s display negative values, thus reduction should infer a dramatic inversion of zeta potentials. Table 2 discloses the obtained results. The original NHGs of 400, 200 and 50 nm disclose negative zeta potentials: −18.6 ± 0.5, −15.4 ± 1.5 and −5.8 ± 0.75 respectively. This is due to the presence of sulfate groups originating from the initiator KPS attached at the extremities of the polymeric chains which are placed in the proximity of the shear plane of the particle. After the reduction process, the zeta potential becomes positive for all the sizes: 22.6 ± 1, 19.2 ± 2 and 11.6 ± 0.55 respectively. The significant rise of zeta potential clearly demonstrates the presence of new free amino groups on the NHG’s which are positively charged at physiological pH. The notable relationship is that heating to 45 °C results in an increased zeta potential for a given NHG’s. This effect can be observed for all the sizes of NHG’s. We attribute this effect to the increased density of negative (before reduction) or positive (after reduction) charges caused by NHG’s shrinking. We propose that the shrunken NHG’s have more negative or positive charges at the shear plane, resulting in a higher charge density.

### 3.3. Fourier-Transform Infrared Spectroscopy (FTIR)

**Figure 3:**
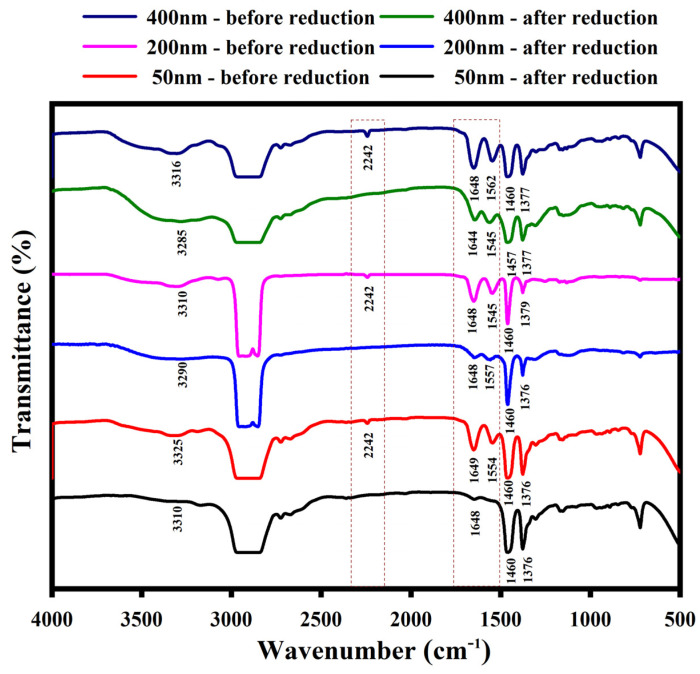
FTIR spectra of the NHG’s (400, 200 and 50 nm).

The FTIR spectra of NHG’s are disclosed in Figure 3. The peak at 2242 cm^−1^ is due to the C-N stretching vibration of the nitrile group in the starting NHG’s. Peaks between 1545 and 1650 cm^−1^ are attributed to the amide (CONH-R) stretching vibration in the starting NHG’s (Navy blue, pink and red plots). Two considerable changes were observed in the FTIR spectra after reduction (green, blue and black plots): (i) the disappearances of C-N stretching vibration due to the formation of reduced NHG’s. (ii) The amide (CONH-R) stretching vibration (1550–1650 cm^−1^) intensities are gradually reduced. The FTIR spectra results clearly confirm the formation of new amino groups originating from nitriles and amides reduction as expected.

In summary, the nitrile groups were fully reduced as the signal at 2242 totally disappeared, the amides were partially reduced the stretching signal are reduced. We assume that the main reduced amides are those of the jeffamine chains since the thermo-responsivity of the NHGs remained like those of the non-reduced NHGs. Together, all the modifications brought about dramatic changes in zeta potentials.

### 3.4. AFM Analysis of NHG’s

**Figure 4:**
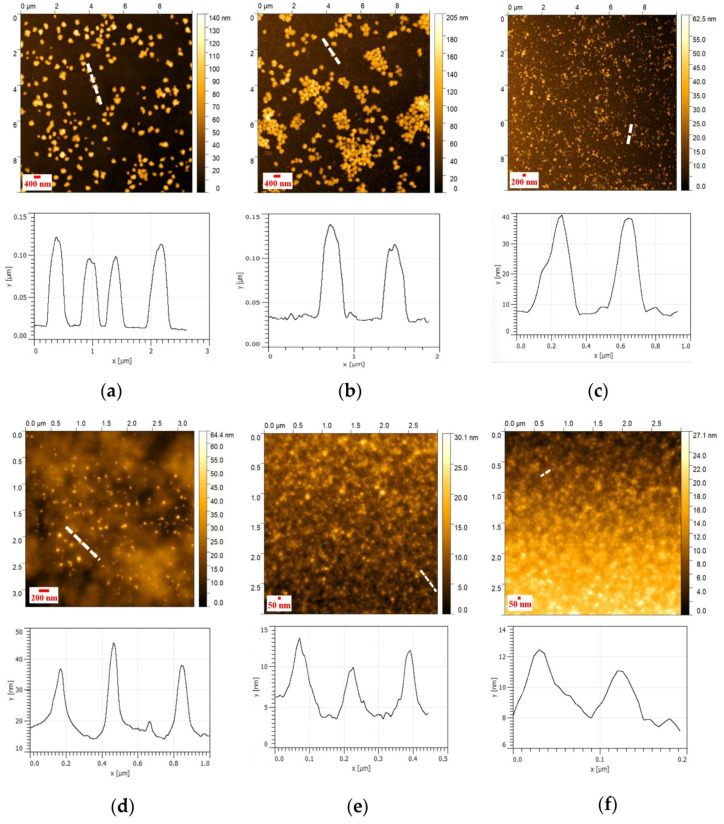
AFM topography images of 400 nm NHG (**a**) original and (**b**) reduced, 200 nm NHG (**c**) original and (**d**) reduced; and 50 nm NHG (**e**) original and (**f**) reduced.

The AFM shows that the size of the dried NHG’s is smaller than the sizes in the water solution as observed by DLS. This is expected due to the dryness of the NHG’s when using the AFM technic. The NHG’s are observed as monodispersed before and after reduction. The topography of NHG’s as observed by AFM indicates that the particle size is monodispersed and spherical in shape (Figure 4).

### 3.5. Complexes of NHG’s with DNA

#### Gel Electrophoresis

**Figure 5:**
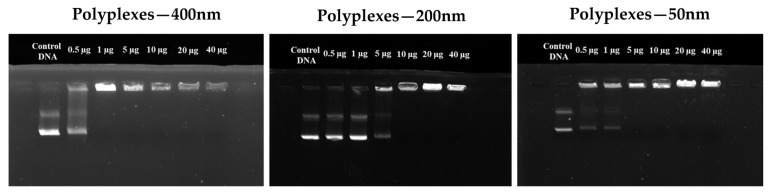
Gel electrophoresis assay with various amounts of reduced NHG’s with GFP plasmid.

Gel electrophoresis (Figure 5) was used to determine the capacity of the NHGs to complex DNA. The experiments were carried out by varying the different concentrations of NHG’s to form a complex with a given amount of DNA. Different amounts of NHG’s (from 0.5 to 40 μg) were used for the analysis and the DNA (0.5 μg) concentration was kept constant in all the experiments. Naked DNA was used as a control. The complexes (polyplexes) of 400, 200, and 50 nm are formed with a strong interaction (non-migration on the gel) starting from 2:1 for 400 nm, 20:1 for 200 nm and 10:1 for 50 nm (NHG’s/DNA) weight ratio. The polyplexes of strong interactions were confirmed by the UV trans-illuminator.

### 3.6. Size and Zeta Potential of Polyplexes

We have prepared NHGs/DNA complexes using 400, 200 and 50 nm NHG’s at different weight ratios (Table 3). The ratios NHG/DNA were from 1:1 to 40:1. Interestingly, we acknowledge that at a low NHG’s ratio complexes are aggregative (AG* in Table 3), while upon arriving at ratio 5:1 the size of complexes become monodispersed around the size of the naked NHG’s. Raising the ratio of NHG’s allows the zeta potential values to increase, at a maximum ratio of 40:1 in 400, 200, and 50 nm to obtain zeta + 22.8, + 18.2 and + 10.9 mV respectively without size changes. The polydispersity indexes were good for 400 and 200 nm complexes but higher for 50 nm complexes. This might indicate that some molecules of plasmid DNA can be partially complexed by 2 NHG’s particles which are too small to complex entire plasmids, as a result of this, we might find some aggregated NHG’s even at positive zeta potential values with an effect on the dispersity of the complex.

#### 3.6.1. AFM of Polyplexes

**Figure 6:**
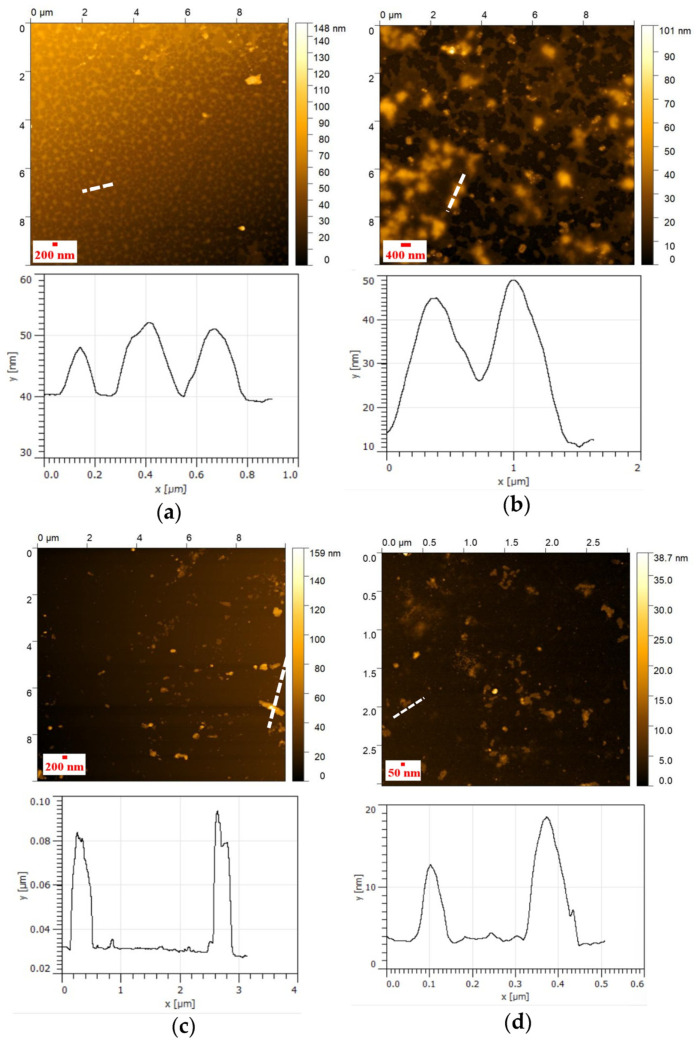
AFM topography images of the DNA and polyplexes ((**a**)—Only pDNA-GFP (0.5 µg), (**b**)—400nm polyplexes, (**c**)—200nm polyplexes and (**d**)—50nm polyplexes). 10 µg of NHG’s and 0.5 µg DNA were used to prepare the polyplex complexes.

The AFM of polyplexes is shown in Figure 6. The size of polyplexes is similar to the size of the reduced NHG’s. NHGs of 400 and 200 nm can efficiently encapsulate the DNA inside the particle. However, two or more particles are needed to complex the DNA for 50 nm NHGs, which causes some aggregation (Figure 6).

#### 3.6.2. Cell Proliferation

**Figure 7:**
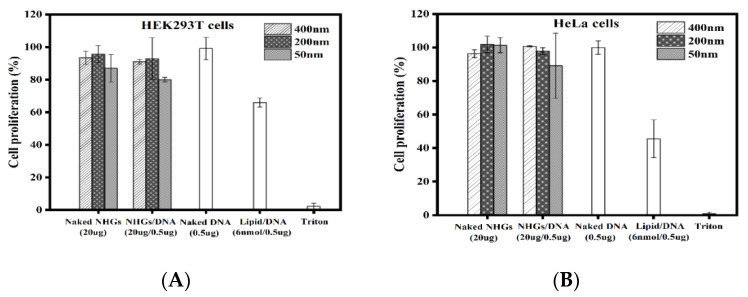
Cell proliferation for the different polyplexes (400, 200 nm and 50 nm) and control lipoplex at 48 h incubation in HEK293 T (**A**) and HeLa (**B**) cell lines. Each point represents the mean value ± SD (n = 2).

The results (Figure 7) demonstrate that polyplexes (400, 200 and 50 nm) are devoid of toxicity using NHGs/DNA ratio of 40:1 at concentrations of 20 µg/0.5 µg, in HEK293T and HeLa cell lines for incubation of 2 days. A standard lipoplex formed with the same DNA (0.5 µg) using cationic lipid RPR- (6 nmol), displayed significant toxicity after 48 h incubation. The cytotoxic effect values of polyplexes were calculated based on quadruplicate at a concentration of 20 µg NHGs/0.5 µg DNA and 20 µg for naked NHGs. The lipopllexes, naked DNA (0.5 µg GFP), and triton were evaluated as controls.

## 4. Conclusions

In this study, we have successfully designed, synthesized, and characterized novel monodispersed nanohydrogels that can be chemically reduced to swap their zeta potential from strong negative to strong positive. The reduced NHGs form complexes with DNA and the obtained complexes are devoid of cell toxicity. The size of the complexes is predetermined by the size of the NHGs; thus, the DNA is fully condensed in the NHGs. Since the new system is not a self-assembly but a polymeric cross-linked nanoparticle with well-defined sizes loaded with DNA, it might be applied to the controlled release of DNA/mRNA for the expression of a foreign protein.

## Figures and Tables

**Table 1:** Sizes of NHG’s as obtained and after reduction. Results are reported as the mean of 3 measurements. All the polymerizations were performed in 10 mL H_2_O containing 170 mg NIPAAM, Acrylonitrile 60 mg (74 µL), 5 mg BIS, 5 mg PVP, and 5 mg K_2_S_2_O_8_ (KPS). (‘‘Block’’ refers to (Acr)1.1 Jeffamine 1900).

#	Amount of Macro Monomer (mg)	Size of NHG’s (nm)							
		Original NHG’s				Reduced NHG’s			
		25 °C	PDI	45 °C	PDI	25 °C	PDI	45 °C	PDI
A	200	401	0.019	371	0.132	407	0.361	361	0.355
B	400	199	0.058	165	0.265	200	0.339	172	0.34
C	500	54	0.028	32	0.33	71	0.372	48	0.362

**Table 2:** Zeta potential value of NHG’s. Results are reported as mean ± S.D (*n* = 3).

#	Size of NHG’s (nm)	ζ (mV)			
		Original NHG’s		Reduced NHG’s	
		25 °C	45 °C	25 °C	45 °C
A	400	−18.6	−20.5	22.6	25
B	200	−15.4	−17	19.2	23
C	50	−5.8	−8.47	11.6	14

**Table 3:** Size and zeta potential of polyplexes (400, 200, and 50 nm). AG*—Aggregation.

#	NHG’s/DNA (wt/wt) Ratio	0:1	1:0	1:10	1:1	5:1	10:1	20:1	40:1
Polyplexes (400 nm NHG)	Size (nm) 25 °C	AG*	405	AG*	AG*	410	394	398	403
	PDI	1.000	0.186	1.000	1.000	0.341	0.242	0.302	0.316
	ζ (mV) 25 °C	−10	22	−3.9	7.1	12.5	13.8	17.7	22.8
Polyplexes (200 nm NHG)	Size (nm) 25 °C	AG*	230	AG*	AG*	255	225	223	227
	PDI	1.000	0.242	1.000	1.000	0.372	0.259	0.267	0.292
	ζ (mV) 25 °C	−8.9	19	−5.7	4.9	10.7	12.4	15.7	18.2
Polyplexes (50 nm NHG)	Size (nm) 25 °C	AG*	62	AG*	AG*	54	65.3	67.6	56
	PDI	1.000	0.772	1.000	1.000	0.942	0.842	0.741	0.686
	ζ (mV) 25 °C	−9.2	12.3	−8.1	3.0	7.3	8.2	10.5	10.9

## Data Availability

Not applicable.

## Abbreviations

ACN	Acrylonitrile
BIS	N, N’-Methylenebisacrylamide
DLS	Dynamic Light Scattering
KPS	Potassium persulfate
NP/NPs	Nanoparticle/ Nanoparticles
NHG’s	Nanohydrogels
NIPAAM	N-Isopropylacrylamide
PDI	Polydispersity index
PEG	Poly (ethylene glycol)
PEO	Poly (ethylene oxide)
PPO	Poly (propylene oxide)
PVP	Polyvinylpyrrolidone
